# Identification and validation of the high expression of pseudogene TCAM1P in cervical cancer via integrated bioinformatics analysis

**DOI:** 10.1186/s12935-021-02440-7

**Published:** 2022-01-11

**Authors:** Yuanhang Zhu, Chenchen Ren, Li Yang, Zhenan Zhang, Meiyuan Gong, Kebing Chen

**Affiliations:** 1 Department of Obstetrics and Gynecology, The Third Affiliated Hospital of Zhengzhou University, Zhengzhou Key Laboratory of Cervical Diseases, No. 7, Front Kangfu Street, Zhengzhou, 450052 Henan People’s Republic of China; 2 Academy of Pharmaceutical Sciences, Zhengzhou University, Zhengzhou, 450052 People’s Republic of China

**Keywords:** Uterine Cervical Neoplasms, Pseudogene, Papillomavirus Infections, EIF4A3 protein

## Abstract

**Background:**

HPV as the main cause of cervical cancer has long been revealed, but the detailed mechanism has not yet been elucidated. The role of testis/cancer antigen in cervical cancer has been revealed. However, there are no reports about the statement of testis/cancer-specific non-coding RNA. In this study, we first proposed TCAM1P as a testis/cancer-specific pseudogene, and used a series of experimental data to verify its relationship with HPV, and analyzed its diagnosis value of high-grade cervical lesions and the mechanism of their high expression in cervical cancer. This provides a new direction for the prevention and treatment of cervical cancer.

**Methods:**

The specific expression of pseudogenes in each tissue was calculated by “TAU” formula. ROC curve was used to judge the diagnosed value of TCAM1P for high-grade lesions. The proliferation ability of cells was measured by CCK8. The expression of TCAM1P, HPV E6/E7 were detected by qRT-PCR. The binding for RBPs on TCAM1P was predicted by starbase v2.0 database, then RIP assay was used to verify. Besides, Gene Ontology (GO) and KEGG enrichment analysis were performed with “clusterprofiler” R package.

**Results:**

TCAM1P was specifically high-expressed in normal testicular tissue and cervical cancer. Interesting, with the severity of cervical lesions increased, the expression of TCAM1P increased, and TCAM1P could effectively diagnose high-grade cervical lesions. Besides, the expression of TCAM1P was HPV dependent, with highest expression in HPV-positive cervical cancer tissues. Furthermore, RIP assay showed that EIF4A3 regulated the expression of TCAM1P through binding with it. CCK8 assay showed that TCAM1P promoted the proliferation and the Gene ontology (GO) and KEGG Pathway enrichment analysis same suggested that TCAM1P is involved in multiple ways in cell proliferation including Cell cycle, DNA replication and etc.

**Conclusions:**

In this study, we firstly proposed that TCAM1P is cancer/testis pseudogene and is regulated by HPV E6/E7 and EIF4A3. TCAM1P promotes the proliferation of cervical cancer cells and acts as promoter in cervical cancer. Otherwise, TCAM1P promote proliferation through regulating cell cycle and DNA replication, but more evidence needs to be provided to reveal the mechanism by which TCAM1P plays a role in cervical cancer.

**Supplementary Information:**

The online version contains supplementary material available at 10.1186/s12935-021-02440-7.

## Background

Cervical cancer (CC) is a rare malignant tumor with a known cause, which provides the foundation for the tertiary prevention of cervical cancer and effectively reduces the incidence and mortality of cervical cancer. But it is undeniable that despite the use of human papilloma virus (HPV) preventive and therapeutic vaccines, cervical cancer has not been completely prevented or treated [1]. The main reason is that the specific mechanism of HPV carcinogenesis is not yet clear. Therefore, on the basis of the currently known causes, further exploration of the pathogenesis of cervical cancer may further prevent the occurrence and improve the survival rate of patients with advanced cervical cancer.

Cancer/testis antigens (CTAs) refers to proteins expressed in several tumors and germ cells of the testis and placenta, but not in other normal tissues, favoring tumor maintenance, proliferation, and metastasis [2]. The role of CTAs in malignant tumor includes as molecular markers, therapeutic targets, etc. [3]. Interesting, at present, the activity of non-coding RNA in tumorigenesis is increasingly revealed, and more and more evidences suggest its important role in tumors [4–7], including the role of pseudogenes in cervical cancer. For example, guanylate binding protein 1 pseudogene 1 (GBP1P1) and pituitary tumor-transforming 3 pseudogene (PTTG3P), were over-expressed in cervical cancer and PTTG3P was positively correlated with PTTG1 levels, which promote cervical cancer cells proliferation through cyclin B1 (CCNB) and increased CC cell invasiveness through upregulation of snail family transcriptional repressor 1 (SNAIL) and downregulation of E-cadherin [8, 9]. Based on this, we speculated whether there are pseudogenes specifically expressed in tumors and testicular tissues, and can be used as screening targets and therapeutic targets.

In this study, we first analyzed whether differentially expressed pseudogenes between cervical cancer and normal cervix were specifically expressed by calculating the tissue specificity index, and identified the specifically and highly expressed pseudogenes, testicular cell adhesion molecule 1 (TCAM1P). Intriguingly, this is the first non-coding RNA called as Cancer/testis pseudogene. Moreover, we verified the relationship between TCAM1P and HPV and confirmed that HPV E6/E7 and EIF4A3 can regulate the expression of TCAM1P, which promoted the proliferation of cervical cancer cells. Moreover, the high diagnostic value of TCAM1P for high-grade cervical lesions suggested that TCAM1P may be a biomarker and therapeutic target for CC treatment.

## Methods

### Identification of cancer/testis (CT) pseudogenes

**Fig. 1:**
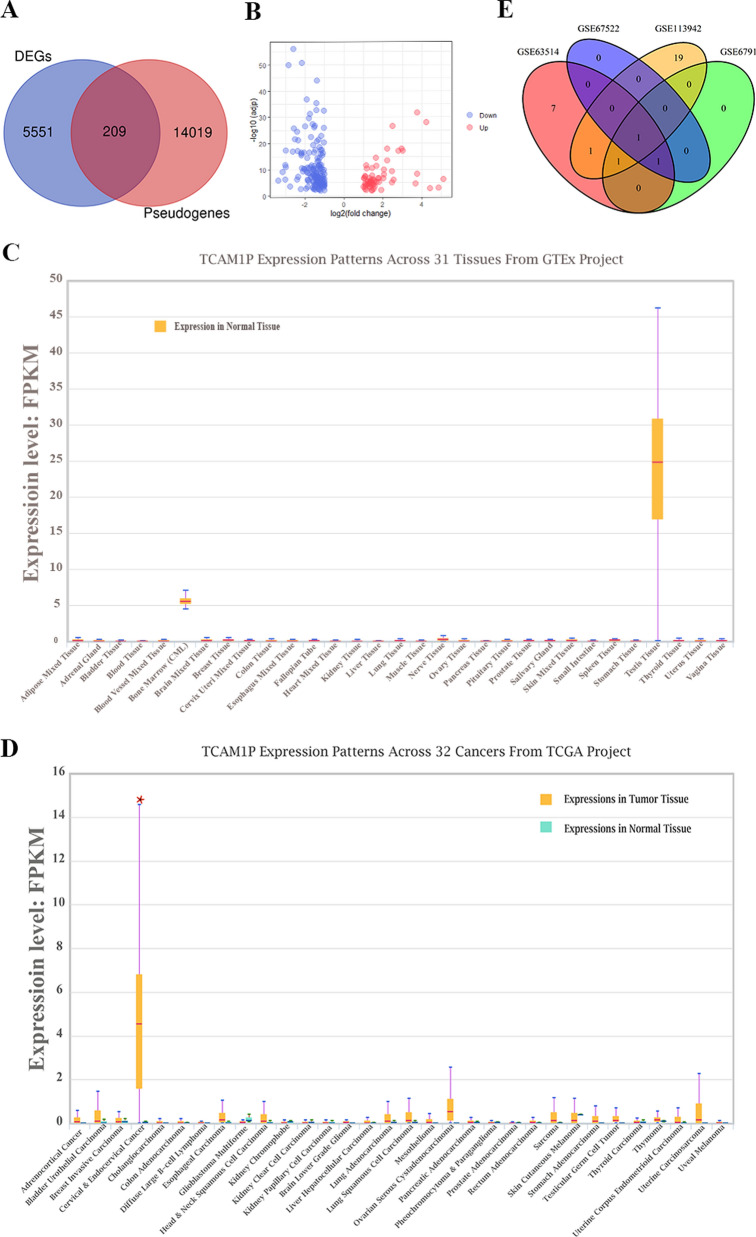
The identification of cancer/testis (CT) pseudogenes TCAM1P in cervical cancer. **A** Co-expression of the differentially expressed genes (DGEs) and pseudogenes by venn analysis. **B** The upregulated and downregulated genes presented by volcano map. **C** Co-expression of the DGEs in different GEO dataset by venn analysis. **D**, **E** The expression of TCAM1P in normal tissues and tumor tissues

Based on the Genotype-Tissue Expression (GTEx) database, we used the TAU formula (Fig. 1) to calculate the *τ* value of each pseudogene, which is best overall method to measure expression specificity [10, 11]. The *τ* value ranges from 0–1, and the closer to 1 indicating that the gene expression in a normal tissue is more specific.

$$ \tau  = \frac{{\sum\nolimits_{{i = 1}}^{n} {\left( {1 - \widehat{{x_{i} }}} \right)} }}{{n - 1}};\quad \widehat{{x_{i} }} = \frac{{x_{i} }}{{\mathop {\max }\limits_{{1 \le i \le n}} {\mkern 1mu} (x_{i} )}}. $$

### Gene expression profile data

In this study, the expression of pseudogenes in cervical cancer and normal cervical tissue were analyzed based on The Cancer Genome Atlas (TCGA) and Gene Expression Omnibus (GEO) database. Data in TCGA were analyzed using an online analysis software, Gene Expression Profiling Interactive Analysis (GEPIA) (http://gepia.cancer-pku.cn/). Data in GEO comes from the following four gene expression profiles (GSE63514, GSE6791, GSE67522 and GSE113942), which were downloaded from the GEO database (http://www.ncbi.nlm.nih.gov/geo). The detailed sample information is presented in Table 1.

**Table 1:** Detailed sample information in GEO databases

GEO database	Group	Number
GSE63514	Normal	24
	CIN 1	14
	CIN 2	22
	CIN 3	40
	Cervical cancer	28
GSE6791	Normal	8
	Cervical cancer	20
GSE67522	Normal	22
	Cervical cancer	20
GSE113942	Normal	7
	Cervical cancer	7

CIN 1, grade 1 cervical intraepithelial neoplasia; CIN 2, grade 2 cervical intraepithelial neoplasia; CIN 3, grade 3 cervical intraepithelial neoplasia

### GO term and KEGG pathway enrichment analysis

After identifying the related genes with TCAM1P, we use the “clusterprofiler” R package [12] to perform Gene Ontology (GO) term and Kyoto Encyclopedia of Genes and Genomes (KEGG) pathway enrichment analysis and to investigate the potential function of these genes to find the biological roles of these related genes.

### Cell culture, cell lines

In this study, we used HCerEpiC (normal cervical epithelial cell line), Hela, Siha, C33a and Caski (these four are cervical cancer cells line). Siha and C33a were obtained from Chinese Academy of Sciences and ATCC, and HCerEpiC, Hela and Caski were donated by Dr Yang Yang who bought from Chinese Academy of Sciences, among them Hela cell was parental sublines. All cells were cultured in Dulbecco's Modified Eagle Medium (DMEM, Boster Biological Technology co., Ltd) with 10% Fetal Bovine Serum (FBS, CLARK, Cat No. FB15015), 100 U/mL penicillin and 100 ug/mL streptomycin (solarbio, cat No. P1400) in 5% CO_2_ at the temperature of 37 °C.

### Cytoplasmic and nuclear RNA purification

In order to identify the main location of TCAM1P in cells, we separately extracted RNA from the nucleus and cytoplasm of cervical cancer cells using the Cytoplasmic and Nuclear RNA purification kit (Norgen Biotek Corp, #21000,37400) according to the manufacturer’s instructions.

### Small interfering RNA (siRNA) interference

To silence the expression of TCAM1P in cells, six interfering sequences targeting TCAM1P were transferred into cells with jet PRIME transfection reagent according to the manufacturer’s instructions. The transfection of small interfering RNA targeting EIF4A3 has been reported in our previous studies [13].

### RNA extraction and Quantitative Real-time PCR (qRT-PCR)

The procedure of total RNA extraction and qRT-PCR has been reported in our previous studies [13]. The primers sequences for qRT-PCR showed in Table 2.

**Table 2:** Sequences of primers

Gene	Sequences
EIF4A3	F: CAACGAGCAATCAAGCAG R: GTGGGAGCCAAGATCAAA
GAPDH	F: TGACTTCAACAGCGACACCCA R: CACCCTGTTGCTGTAGCCAAA
TCAM1P	F: GTTAGAGGAATCCAGTTGCCCT R: GTTGCCTTCTGTGGCACTTC
HPV 16 E6/E7	F: CAATGTTTCAGGACCCACAGG R: CTCACGTCGCAGTAACTGTTG
HPV 18 E6/E7	F: ATGCATGGACCTAAGGCAAC R: AGGTCGTCTGCTGAGCTTTC

### Western blot assay

RIPA lysis buffer (cat# R0020; Solarbio, Beijing, China) and BCA Protein Assay kit (PA101-01, Biomed, Beijing, China) were used to complete protein extraction and protein concentration measurement. Antibodies used in this study included anti-P53 (1:1000 dilution, Abcam, ab183544), anti-GAPDH (1: 5000 dilution, bioss, bs-2188R).

### Cell Counting Kit-8 (CCK-8) assay

The proliferation ability of treated Siha and Caski cells (2 × 10^3^ cells/well) were detected by CCK-8 (Dojindo Laboratories, Kumamoto, Japan) after 0, 24, 48, 72 and 96 h of growth and measure the absorbance at 450 nm.

### Colony formation assay

The treated Siha and Caski cells (1000 cells/well) were seeded into 6-well plate. After 2 weeks, we observed the formation of clone number and stained with Giemsa dilution (Cat. # G1010, Solarbio, Beijing, China).

### RNA binding protein immunoprecipitation (RIP) assay

The procedure of RIP assay was described in our previous studies [13]. The precipitated RNA was measured by qRT-PCR.

### Statistical analysis

Statistical comparison was performed through Student’s t-test or nonparametric test between two groups. Receiver operating characteristic (ROC) curve was drew to assess the optimal diagnosis of TCAM1P. Two-sided P value < 0.05 was considered statistically significant. All statistical analysis was performed by using SPSS (version 24) and GraphPad Prism (version 8.02).

## Results

### The identification of cancer/testis (CT) pseudogenes TCAM1P in cervical cancer

First, we screened out all pseudogenes based on the current human pseudogene annotation in HUGO gene nomenclature committee (HGNC, https://www.genenames.org/). A total of 14,228 genes are defined as pseudogenes. Next, we screened the differentially expressed genes (DEGs) between cervical cancer tissues and matched normal tissues by using GEPIA. 5760 DEGs were screened out. Venn analysis was conducted to determine the DEPGs between cervical cancer tissues and normal cervical tissues. A total of 209 pseudogenes were identified, among which 59 pseudogenes up-regulated and 150 down-regulated pseudogenes (Additional file 1, Fig. 1A, B).

In order to screen out CT pseudogenes from the 209 DEPGs, we downloaded the expression levels of these DEPGs in normal tissues from the GTEx database, and calculated the Tau index of each pseudogene (Additional file 2). Judging from these data, we can see that pseudogenes of TCAM1P, CDC28 protein kinase regulatory subunit 1B pseudogene 3 (CKS1BP3), transmembrane protein 191A (pseudogene) (TMEM191A), ubiquitin A-52 residue ribosomal protein fusion product 1 pseudogene 6 (UBA52P6), POM121 transmembrane nucleoporin like 9, pseudogene (POM121L9P), ATP binding cassette subfamily A member 17, pseudogene (ABCA17P), MSL complex subunit 3 pseudogene 1 (MSL3P1), ubiquitin conjugating enzyme E2 S pseudogene 1 (UBE2SP1), zinc finger DHHC-type palmitoyltransferase 8 pseudogene 1(ZDHHC8P1) were potential CT pseudogenes because the Tau index of them greater than 0.95, especially for TCAM1P (Tau index higher than 0.99). The expression of TCAM1P in different normal tissue and different cancer were showed in Fig. 1C, D, which showed that TCAM1P is mainly expressed in testicular tissue but TCAM1P expression is increased in cervical cancer.

To further confirm that TCAM1P is highly expressed in cervical cancer, public datasets available from GEO were mined to compare the expression level of TCAM1P in cervical cancer and matched normal tissue. Results showed that only TCAM1P is the common DEPGs (Fig. 1E) and the expression level of TCAM1P in cervical cancer is higher than normal cervix tissue in all datasets that detect TCAM1P (Table 3).

**Table 3:** The differential expression of TCAM1P in different datasets

GEO database	LogFC	Adj. P value
GSE63514	4.64	2.06E−08
GSE6791	2.61	9.33E−05
GSE67522	2.75	2.76E−09
GSE113942	8.13	3.69E−15

### The relationship between TCAM1P and HPV infection in patients with CC

**Fig. 2:**
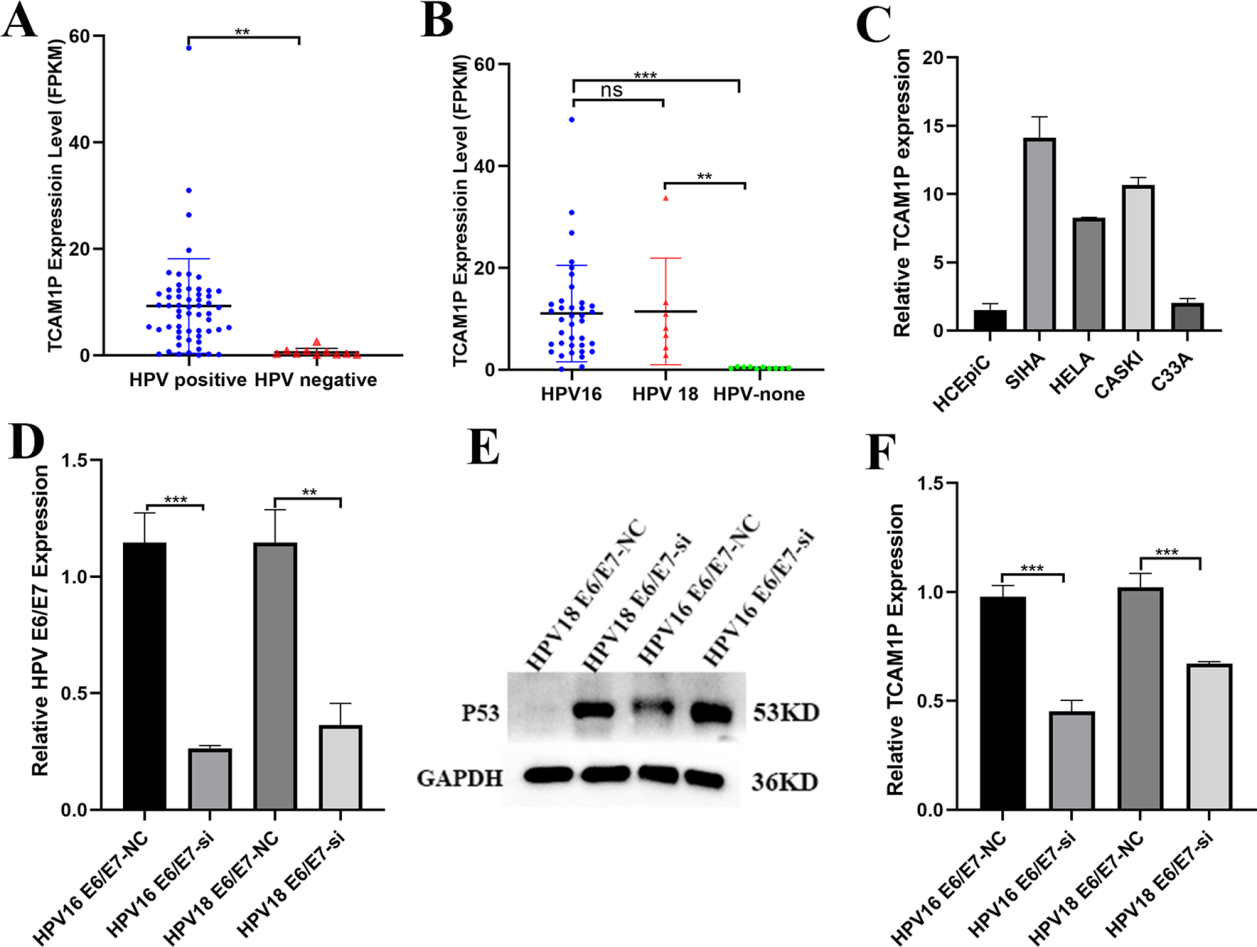
The relationship between TCAM1P and HPV infection in patients with CC. **A**–**C**, the expression of TCAM1P were evaluated by qRT-PCR assay. **D** the expression of E6/E7 were evaluated by qRT-PCR assay. **E** WB assay was used to detected the expression of P53. **F** the expression of TCAM1P were evaluated by qRT-PCR assay. The data are presented as the mean ± SD, **P < 0.01, ***P < 0.001

HPV infection is the main cause of cervical cancer. Therefore, we look for evidence to explore whether the high expression of TCAM1P is related to HPV infection. First, we compare the expression level of TCAM1P between HPV-positive cervical cancer and HPV-negative cervical cancer based on GEO datasets. Results showed that there was a difference in the expression of TCAM1P between the two groups according to GSE151666 (Fig. 2A). Considering that HPV 16 and HPV 18 were the most common and most carcinogenic infection type, we further analyzed whether there is a differential expression of TCAM1P in HPV16/18-positive cervical cancer and HPV-negative cervical cancer. Results showed that the expression of TCAM1P in HPV16/18-positive cervical cancer is higher than in HPV-negative cervical cancer (Fig. 2B). In order to further verify that the expression of TCAM1P is related to HPV infection, we used RT-PCR to detect the expression of TCAM1P in different cervical cancer cells. Results showed that TCAM1P expression in Siha and Caski is Highest, followed by Hela and lowest in C33a, which means that TCAM1P is HPV-dependent (Fig. 2C). The HPV E6/E7 are the major oncoproteins in cervical cancer. To detect whether the expression of TCAM1P is related to HPV E6/E7 protein, we tested the expression level of TCAM1P when HPV16 E6/E7 (Siha) and HPV18 E6/E7 (Hela) are knocked down (Fig. 2D, E). Results showed that the expression of TCAM1P has decreased when HPV E6/E7 are knocked down (Fig. 2F).

### Correlation between TCAM1P and clinicopathological characteristics in patients with CC

**Fig. 3:**
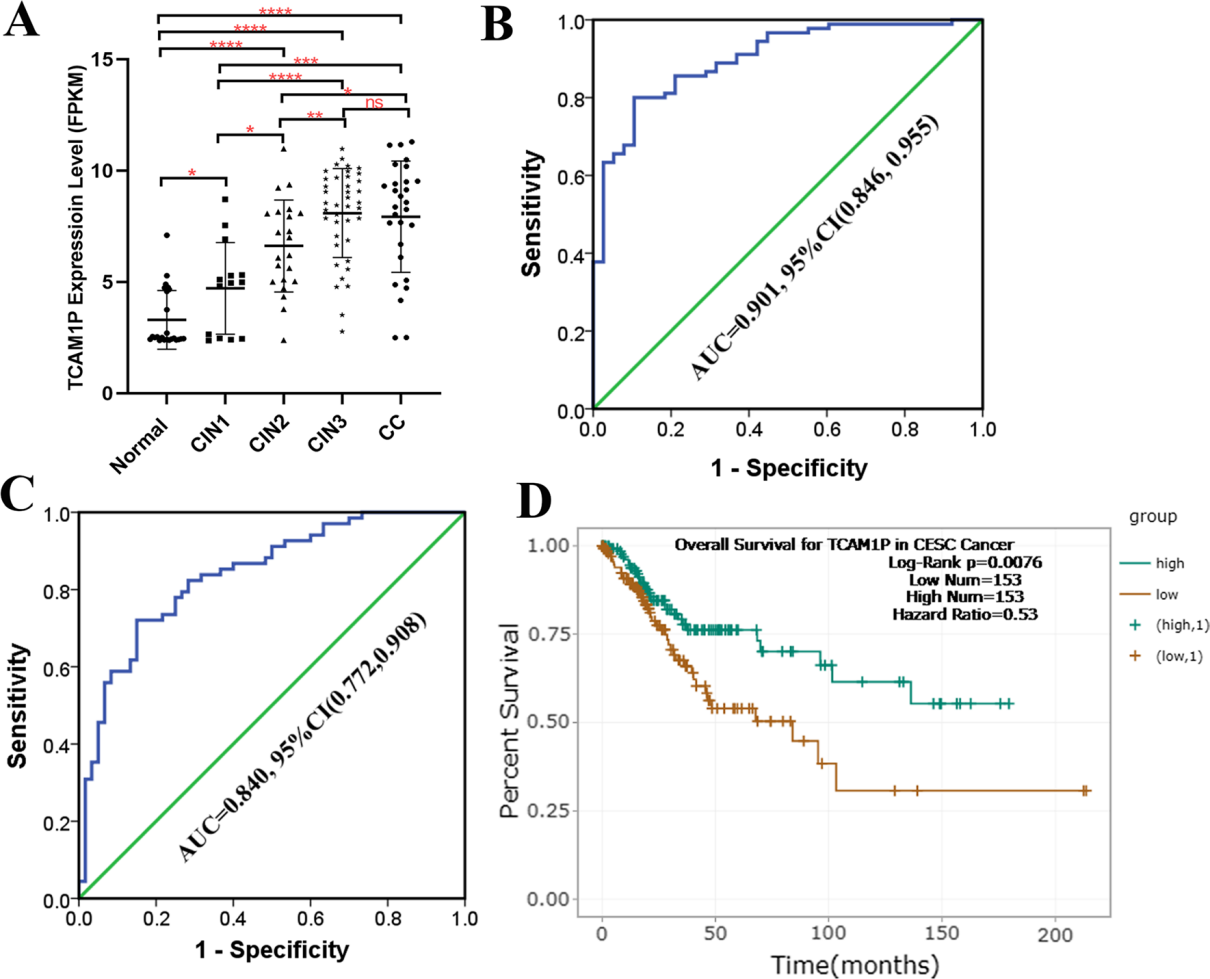
Correlation between TCAM1P and clinicopathological characteristics in patients with CC. **A** the expression of TCAM1P in different cervical lesions. **B** The value of TCAM1P in the diagnosis of CIN2 and above lesions detected by ROC curve. **C** The value of TCAM1P in the diagnosis of CIN3 and above lesions detected by ROC curve. **D** KM curves depicting OS for CC patients with high and low expression of TCAM1P. The data are presented as the mean ± SD, *P < 0.05, **P < 0.01, ***P < 0.001, ****P < 0.0001

Considering that HPV infection is related to the elevated expression of TCAM1P in the above-mentioned consolidated cervical cancer, we further explored whether TCAM1P is related to the level of cervical lesions. First, we analyzed the expression of TCAM1P in different cervical lesion levels based on GSE63514. Results showed that with the level of cervical lesions increased, the expression of TCAM1P increased (Fig. 3A). ROC curve was used to detect the diagnostic value of TCAM1P for high-grade cervical lesions based on data in GSE63514. Results showed that TCAM1P can accurately diagnose high-grade cervical lesions with area under the curve (AUC) greater than 0.7 (Fig. 3B, C). Next, we analyzed the relationship between TCAM1P expression and clinicopathological characteristics based on TCGA database. Result showed that TCAM1P expression is higher in patients with Lymph node metastasis than in non-metastasis while the difference was not statistically significant in the remaining groups (Table 1). Otherwise, we also found that the TCAM1P expression difference in patients with P53 mutation or not existed. Survival analysis results showed that higher TCAM1P is a protective factor for patients with cervical cancer (Fig. 3D).

### TCAM1P promotes the proliferation of cervical cancer cells

**Fig. 4:**
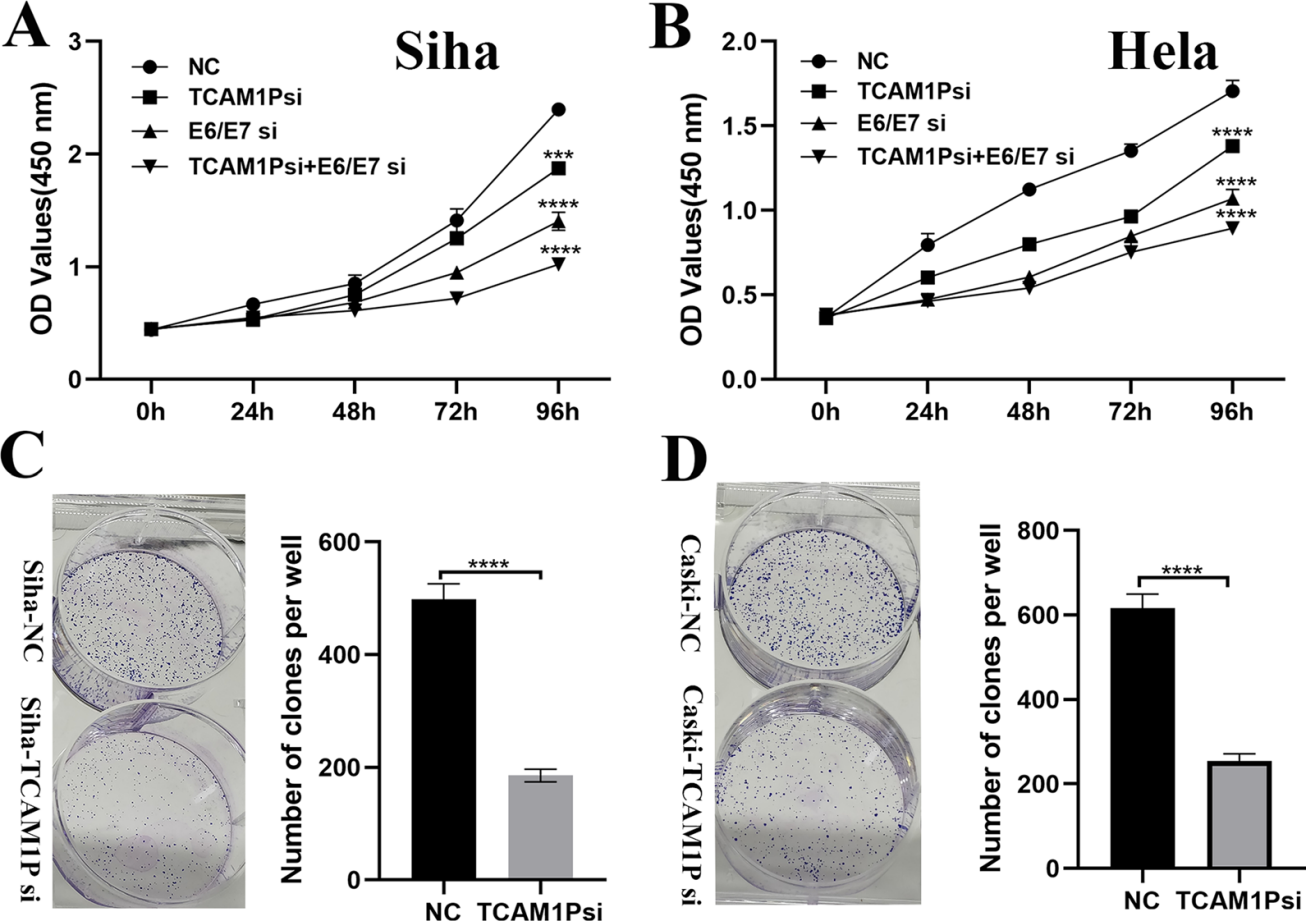
TCAM1P promotes the proliferation of cervical cancer cells. **A**, **B**, Cell proliferation abilities were detected by CCK-8 for treated Siha and Caski cells. **C**, **D**, Cell proliferation abilities were detected by colony formation assays for treated Siha and Caski cells. The data are presented as the mean ± SD, *P < 0.05, **P < 0.01, ***P < 0.001, ****P < 0.0001

Considering that the expression of TCAM1P in HPV16-positive cervical cancer cells is higher than that in HPV18-positive and HPV-negative cervical cancer cells, we used Siha and Caski cells to silence TCAM1P, and then used CCK8 and clone formation experiments to analyze the effects of TCAM1P on cell proliferation functions. Results showed that silencing of TCAM1P significantly inhibited the proliferation ability of Siha and Caski cells, and silence of HPV 16 E6/E7 further inhibited cell proliferation (Fig. 4A, B). Clone formation assay also showed that silencing of TCAM1P suppressed cell proliferation (Fig. 4C, D).

### EIF4A3 promotes the proliferation of cervical cancer cells by regulating the expression of TCAM1P

**Fig. 5:**
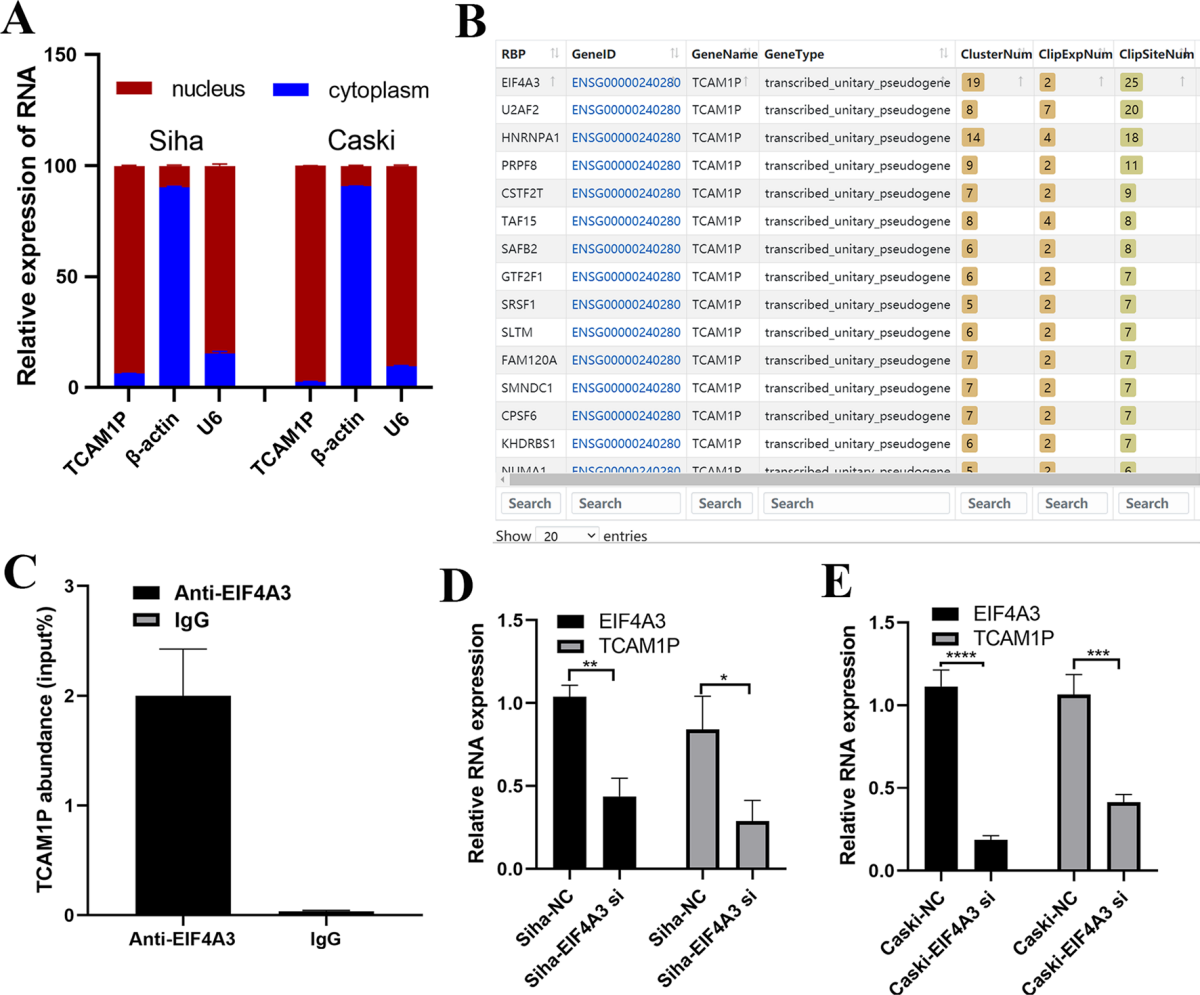
EIF4A3 promotes the proliferation of cervical cancer cells by regulating the expression of TCAM1P. **A** The expression of TCAM1P in nucleus and cytoplasm. **B** The RNA binding proteins (RBPs) that binds to TCAM1P analyzed by StarBase. **C** qRT-PCR was used to detect the transcript abundance relative to input. D, E, Siah and Caski cells were transfected with control or EIF4A3 siRNA, and expression of EIF4A3 and TCAM1P was detected by qRT-PCR. The data are presented as the mean ± SD, *P < 0.05, **P < 0.01, ***P < 0.001, ****P < 0.0001

To clarify the mechanism of high expression of TCAM1P in cervical cancer, we detected the expression of TCAM1P in the cytoplasm and nucleus. Results showed that TCAM1P is mainly expressed in nucleus (Fig. 5A). RNA-binding proteins (RBPs) is widely involved in RNA metabolism process [14]. By using bioinformatics analysis software (https://starbase.sysu.edu.cn/), we found that many RBPs have binding sites with TCAM1P. EIF4A3 attracted our attention because it has most binding sites with TCAM1P (Fig. 5B), which was detected by High Throughput Sequencing after in vivo Crosslinking and Immunoprecipitation (HITS-CLIP) assay. In order to further confirm that there is a combination between the TCAM1P and EIF4A3, RIP assay was conducted by using EIF4A3 antibody. Results showed that TCAM1P does exist among RNAs binding to EIF4A3 (Fig. 5C). Then, we knocked down EIF4A3 using siRNA and found that the expression of TCAM1P was significantly reduced, which means that EIF4A3 regulated the expression of TCAM1P (Fig. 5D, E).

### The function enrichment analysis of TCAM1P related genes

**Fig. 6:**
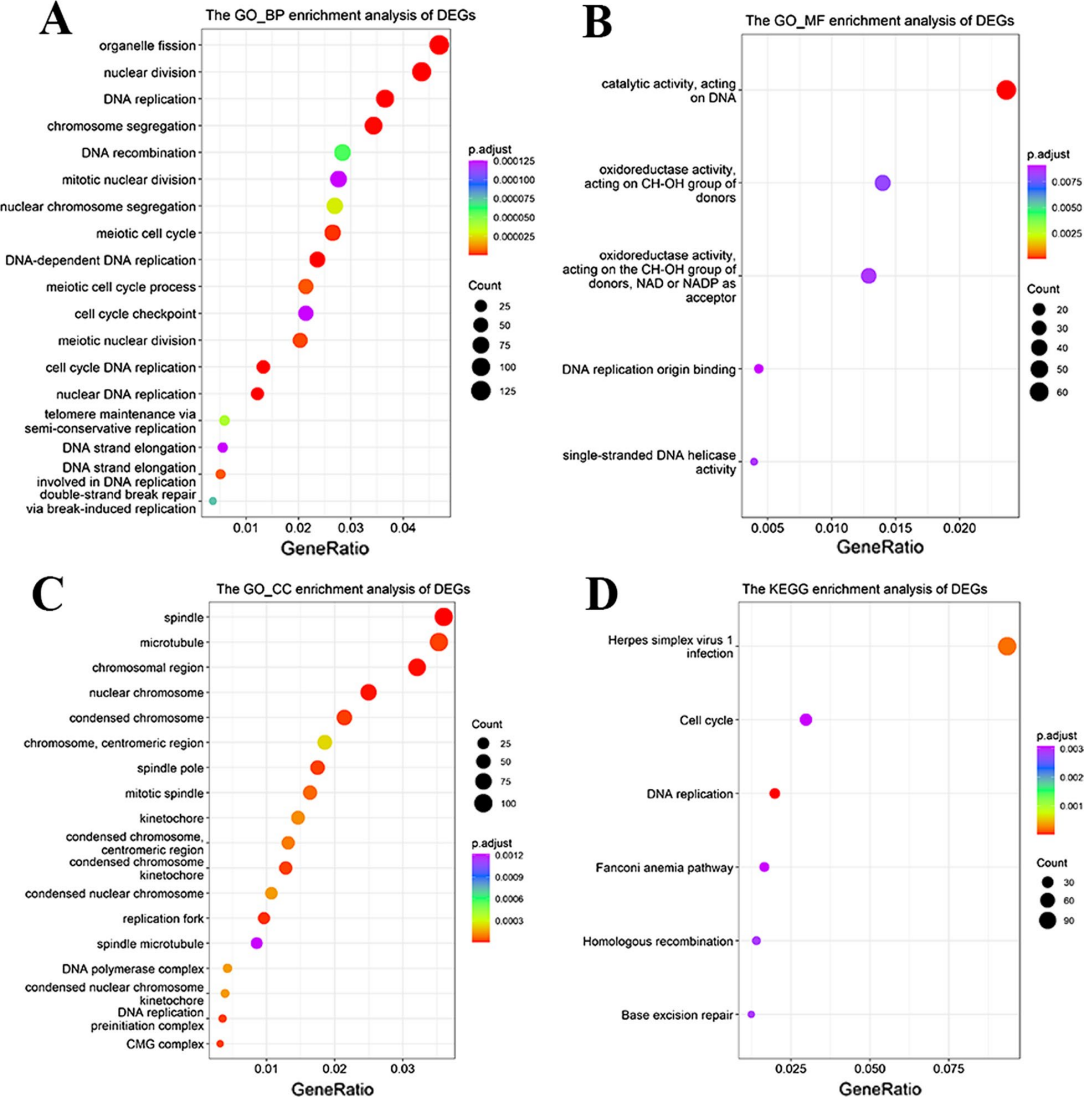
The function enrichment analysis of TCAM1P related genes. **A** Biological processes (BP), **B** molecular function (MF), **C** cell component (CC), **D** KEGG pathway analyses. The x-axis represents the q value (− log10), and the y-axis represents the GO term. The GO terms were measured by the rich factor, q value and number of genes enriched. The greater the Rich factor is, the greater the degree of enrichment and the greater the p value [0, 1]. The brighter the color of red is, the more significant the term

To further clarify the downstream mechanism of TCAM1P, based on the TCGA database, we screened out the proteins related to TCAM1P, and performed GO and KEGG enrichment analysis on them. Results showed that related genes were mainly involved in the biological processes (BP) such as “organelle fission”, “nuclear division” and “DNA replication”, etc. (Fig. 6A). For molecular function (MF), these genes showed enrichment in “catalytic activity, acting on DNA”, “oxidoreductase activity, acting on CH-OH group of donors” and “single-stranded DNA helicase activity” etc. (Fig. 6B). Besides, cell component (CC) enriched predominantly at “spindle”, “microtubule” and “chromosomal region”, etc. (Fig. 6C).

KEGG pathway analysis showed that six significant enrichment pathways existed, including “Herpes simplex virus 1 infection” “Cell cycle,” “DNA replication” “Fanconi anemia pathway,” “Homologous recombination” and “Base excision repair” (Fig. 6D).

## Discussion

Cervical cancer, as a tumor unique to women, seriously endangers women's fertility and life. Therefore, if genes specifically expressed in male testicular tissues have been found to be highly expressed in cervical cancer tissues, and they will be expected to become specific markers for cervical cancer screening. Therefore, in this study, we are committed to screening testicular tissue-specific genes specifically high expressed in cervical cancer tissue. Finally, TCAM1P was found to meet the above condition.

When comprehensively analyzing the expression of TCAM1P in all cancers based on TCGA database, we found that the expression of TCAM1P in cervical cancer, acute myeloid leukemia and Testicular Germ Cell Tumors are increased, which means that TCAM1P may participate in tumorigenesis through the common pathway of cancer. However, Xu et al. found that downregulation of TCAM1P was frequently observed in hepatocellular carcinoma (HCC) compared with adjacent normal tissues and higher TCAM1P expression was related to longer overall survival of patients with HCC. In vivo and in vitro experiments found that TCAM1P inhibited the proliferation of cancer cells through interacted with insulin like growth factor 2 mRNA binding protein 1 (IGF2BP1) and H1.2 linker histone, cluster member (HIST1H1C) and promoted DNA damage inducible transcript 3 (DDIT3) expression in HCC [15]. To further determine the role of TCAM1P in cervical cancer, we once again analyzed its expression level in cervical cancer based on multiple GEO data sets and found that its expression in cervical cancer was higher than that in normal cervical tissue. Cell function testing found that silenced the expression of TCAM1P in cervical cancer cell, the proliferation ability is significantly reduced. Considering this, we believe that TCAM1P acts as a promoter in tumorigenesis of cervix.

HPV is the main cause of cervical cancer. Scholars have found that it can regulate the expression of non-coding RNA and promote the occurrence of cervical cancer [16]. HPV E6/E7 was found to regulate the expression of multiple lncRNAs, such as Pvt1 oncogene (PVT1), metastasis associated lung adenocarcinoma transcript 1 (MALAT1), small nucleolar RNA host gene 12 (SNHG12), lnc-CCDST, LINC01101 and LINC00277 [17]. In our study, we also found that the expression of TCAM1P in HPV-positive cervical cancer is higher than HPV-negative cervical cancer at the tissue and cell levels. When silenced the expression of HPV E6/E7 by using siRNA, TCAM1P expression is reduced. These results showed that the expression of TCAM1P is HPV-dependent. Otherwise, RBPs are reported to be widely involved in the synthesis and stabilization of mature RNA. In our study, we found that EIF4A3 regulated the expression of TCAM1P, which means high expression of TCAM1P in cervical cancer is regulated by EIF4A3. It just so happens that our previous data does show that EIF4A3 is involved in the occurrence of cervical cancer [18].

Cancer/testis antigen and non-coding RNA has shown amazing potential clinical application value in early tumor screening and targeted therapy [19–23]. TCAM1P acted as cancer/testis specific pseudogene need more data to explore its value. Cervical cancer is a gradual disease, and it can be effectively and completely cured for cervical intraepithelial lesions. In this study, we found that the AUC of TCAM1P for CIN 2 or CIN3 are as high as 0.901 or 0.840, which means that TCAM1P can effectively diagnose high-grade cervical lesions and can be an effective method for cervical cancer screening. Further, we found that the expression of TCAM1P is higher in cervical cancer with lymph node metastasis or P53-mutation than without. The former means that TCAM1P is related to Severity of cervical cancer. The latter means that TCAM1P may be related to the choice of drugs targeting P53. This suggested the potential clinical value of TCAM1P in cervical cancer screening and targeted therapy, but more evidence is needed. However, when we analyzed the prognostic value of TCAM1P in cervical cancer, we found that high expression of TCAM1P is related to good prognosis. This is contrary to our research results which may be due to factors such as age, the level of pathological changes of cervical cancer, and differences in treatment options and, etc. Therefore, more work needs to be done to truly reveal the role of TCAM1P in the prognosis of cervical cancer.

TCAM1P lost its original functions in the process of evolution, and is expressed as non-coding RNAs [24]. In order to explore its new function in human cancer. The proteins related to TCAM1P were analyzed through GO function enrichment analysis. The function of related genes is extensively enriched in cell proliferation, such as organelle fission, nuclear division and DNA replication and, ect and enriched pathway are also related to cell proliferation, such as cell cycle and DNA replication. One enriched pathway attracts our attention, herpes simplex virus 1 infection. Herpes simplex virus is DNA virus which is similar with HPV. Therefore, we speculated that TCAM1P may have the potential of regulating HPV virus, but our data didn’t show that TCAM1P regulated the expression of HPV E6E7 (Additional file 3). We think that there may be the possibility of TCAM1P regulating HPV in other ways, which requires us to provide further data to clarify this phenomenon.

## Conclusions

Thus, in this study, we firstly proposed that TCAM1P is cancer/testis (CT) pseudogenes and is regulated by HPV E6/E7 and EIF4A3. TCAM1P promotes the proliferation of cervical cancer cells and acts as promoter in cervical cancer. Otherwise, TCAM1P promoted proliferation through regulating cell cycle and DNA replication, but more evidence needs to be provided to reveal the mechanism by which TCAM1P plays a role in cervical cancer.

## Supplementary Information

**Additional file 1. **All differentially expressed pseudogenes in cervical cancer tissues and normal cervical tissues.

**Additional file 2. **The Tau index of each differentially expressed pseudogenes.

**Additional file 3. **The expression of HPV 16 E6/E7 mRNA.

## Data Availability

The datasets used and/or analyzed during the current study are available from the corresponding author on reasonable request.

## Abbreviations

CC: Cervical cancer
HPV: Human papilloma virus
CTAs: Cancer/testis antigens
GBP1P1: Guanylate binding protein 1 pseudogene 1
PTTG3P: Pituitary tumor-transforming 3 pseudogene
CCNB: Cyclin B1
SNAIL: Snail family transcriptional repressor 1
TCAM1P: Testicular cell adhesion molecule 1
CT: Cancer/testis
GTEx: Genotype-Tissue Expression
TCGA: The Cancer Genome Atlas
GEO: Gene Expression Omnibus
GEPIA: Gene Expression Profiling Interactive Analysis
CIN 1: Grade 1 cervical intraepithelial neoplasia
CIN 2: Grade 2 cervical intraepithelial neoplasia
CIN 3: Grade 3 cervical intraepithelial neoplasia
GO: Gene Ontology
KEGG: Kyoto Encyclopedia of Genes and Genomes
DMEM: Dulbecco's Modified Eagle Medium
SiRNA: Small interfering RNA
EIF4A3: Eukaryotic Translation Initiation Factor 4A3
qRT-PCR: Quantitative Real-time PCR
CCK-8: Cell Counting Kit-8
RIP: RNA binding protein immunoprecipitation
ROC: Receiver operating characteristic
HGNC: HUGO gene nomenclature committee
DEGs: Differentially expressed genes
CKS1BP3: CDC28 protein kinase regulatory subunit 1B pseudogene 3
TMEM191A: Transmembrane protein 191A (pseudogene)
UBA52P6: Ubiquitin A-52 residue ribosomal protein fusion product 1 pseudogene 6
POM121L9P: POM121 transmembrane nucleoporin like 9, pseudogene
ABCA17P: ATP binding cassette subfamily A member 17, pseudogene
MSL3P1: MSL complex subunit 3 pseudogene 1
UBE2SP1: Ubiquitin conjugating enzyme E2 S pseudogene 1
ZDHHC8P1: Zinc finger DHHC-type palmitoyltransferase 8 pseudogene 1
RBPs: RNA-binding proteins
EIF4A3: Eukaryotic Translation Initiation Factor 4A3
HITS-CLIP: High Throughput Sequencing after in vivo Crosslinking and Immunoprecipitation
BP: Biological processes
MF: Molecular function
CC: Cell component
HCC: Hepatocellular carcinoma
IGF2BP1: Insulin like growth factor 2 mRNA binding protein 1
HIST1H1C: H1.2 linker histone, cluster member
DDIT3: DNA damage inducible transcript 3
PVT1: Pvt1 oncogene
MALAT1: Metastasis associated lung adenocarcinoma transcript 1
SNHG12: Small nucleolar RNA host gene 12
