# Correction to: Identification and validation of the high expression of pseudogene TCAM1P in cervical cancer via integrated bioinformatics analysis

**DOI:** 10.1186/s12935-022-02516-y

**Published:** 2022-02-12

**Authors:** Yuanhang Zhu, Chenchen Ren, Li Yang, Zhenan Zhang, Meiyuan Gong, Kebing Chen

**Affiliations:** 1 Department of Obstetrics and Gynecology, The Third Affiliated Hospital of Zhengzhou University, Zhengzhou Key Laboratory of Cervical Diseases, No. 7, Front Kangfu Street, Zhengzhou, 450052 Henan People’s Republic of China; 2 Academy of Pharmaceutical Sciences, Zhengzhou University, Zhengzhou, 450052 People’s Republic of China

## Correction to: Cancer Cell International (2022) 22:17 https://doi.org/10.1186/s12935-021-02440-7

In this article [1], there was an error in naming the Table 4. In the paragraph, Results of "Correlation between TCAM1P and clinicopathological characteristics in patients with CC", the sentence "Result showed that TCAM1P expression is higher in patients with Lymph node metastasis than in non-metastasis while the difference was not statistically significant in the remaining groups (Table 1)’’. This should be Table 4. Also, in Fig. 4B, it is not “Hela” but “Caski”.

 Table 4 and corrected Fig. 4 are given in this erratum.

**Fig. 4:**
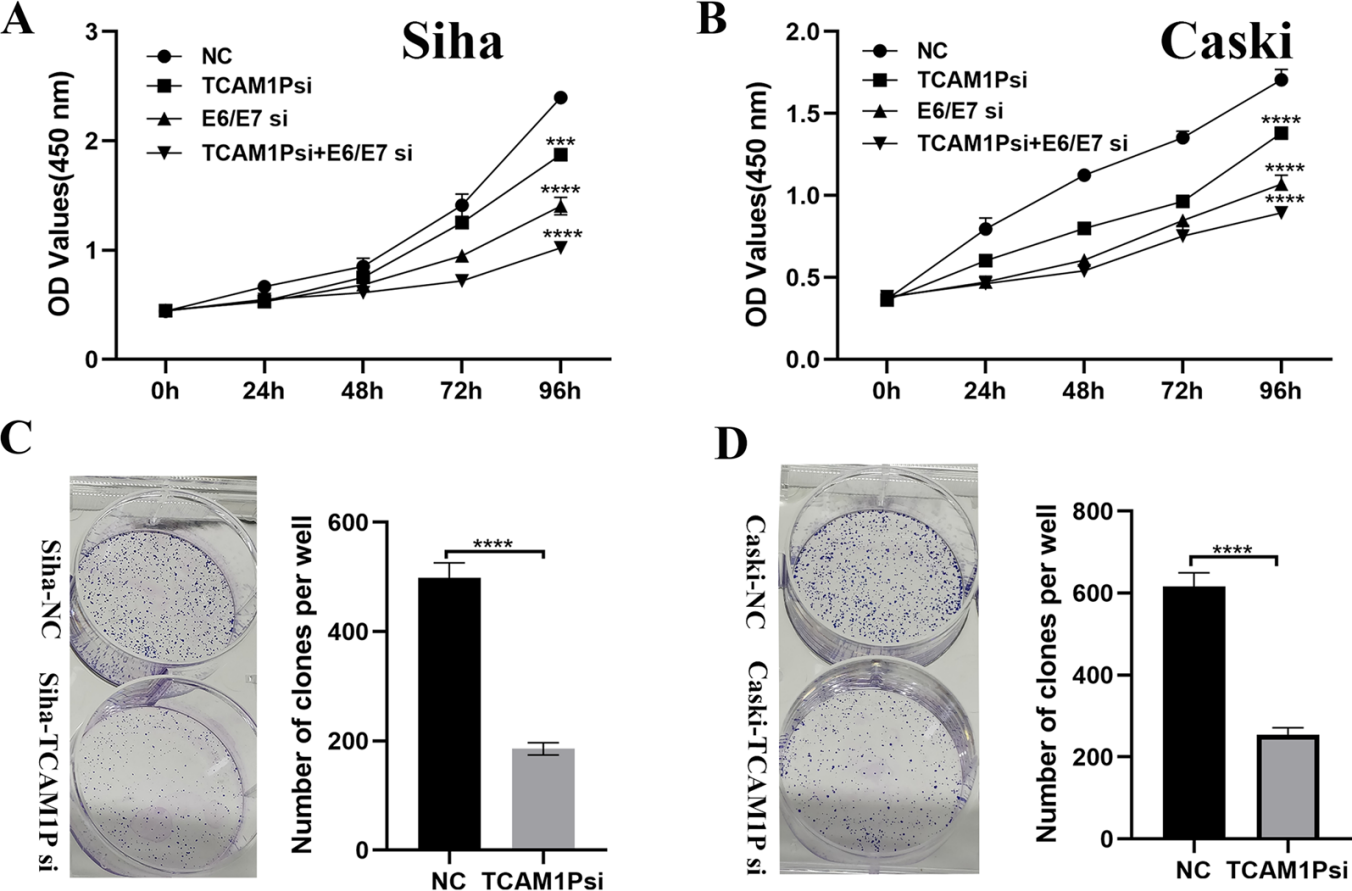
TCAM1P promotes the proliferation of cervical cancer cells. **A**, **B** Cell proliferation abilities were detected by CCK-8 for treated Siha and Caski cells. **C**, **D** Cell proliferation abilities were detected by colony formation assays for treated Siha and Caski cells. The data are presented as the mean ± SD,  ***P < 0.001, ****P < 0.0001

**Table 4:** Analysis of the relationship between the expression of TCAM1P and the clinicopathological characteristics of cervical cancer patients based on the TCGA database

Characteristics	Number	TCAM1 expression	t value	P value
Age (years)			**0.927**	**0.355**
< 40	84	8.37 ± 2.77		
≥ 40	220	8.01 ± 3.04		
Figo stage			**−** **0.667**	**0.505**
I–II	231	8.05 ± 3.00		
III–IV	66	8.32 ± 2.87		
TNM stage			**0.084**	**0.933**
T1–T2	212	8.08 ± 3.01		
T3–T4	30	8.03 ± 3.12		
Histological type			**1.012**	**0.316**
Squamous cell carcinoma	252	8.23 ± 2.81		
Adenocarcinoma	48	7.69 ± 3.51		
Lymph nodes metastasis			**−** **2.896**	**0.004**
Yes	60	8.94 ± 2.08		
No	133	7.82 ± 3.16		
Distant metastasis			**0.951**	**0.343**
Yes	10	6.91 ± 4.32		
No	116	7.92 ± 3.13		
TP53 mutation			**2.612**	**0.016**
Mutation	22	5.97 ± 3.95		
Wide type	287	8.21 ± 2.88		
