# Supplementary figures and images for: Identification and validation of the high expression of pseudogene TCAM1P in cervical cancer via integrated bioinformatics analysis
Source: Cancer Cell Int. 2022 Jan 11;22:17. doi: 10.1186/s12935-021-02440-7 (PMC8753837; doi:10.1186/s12935-021-02440-7)

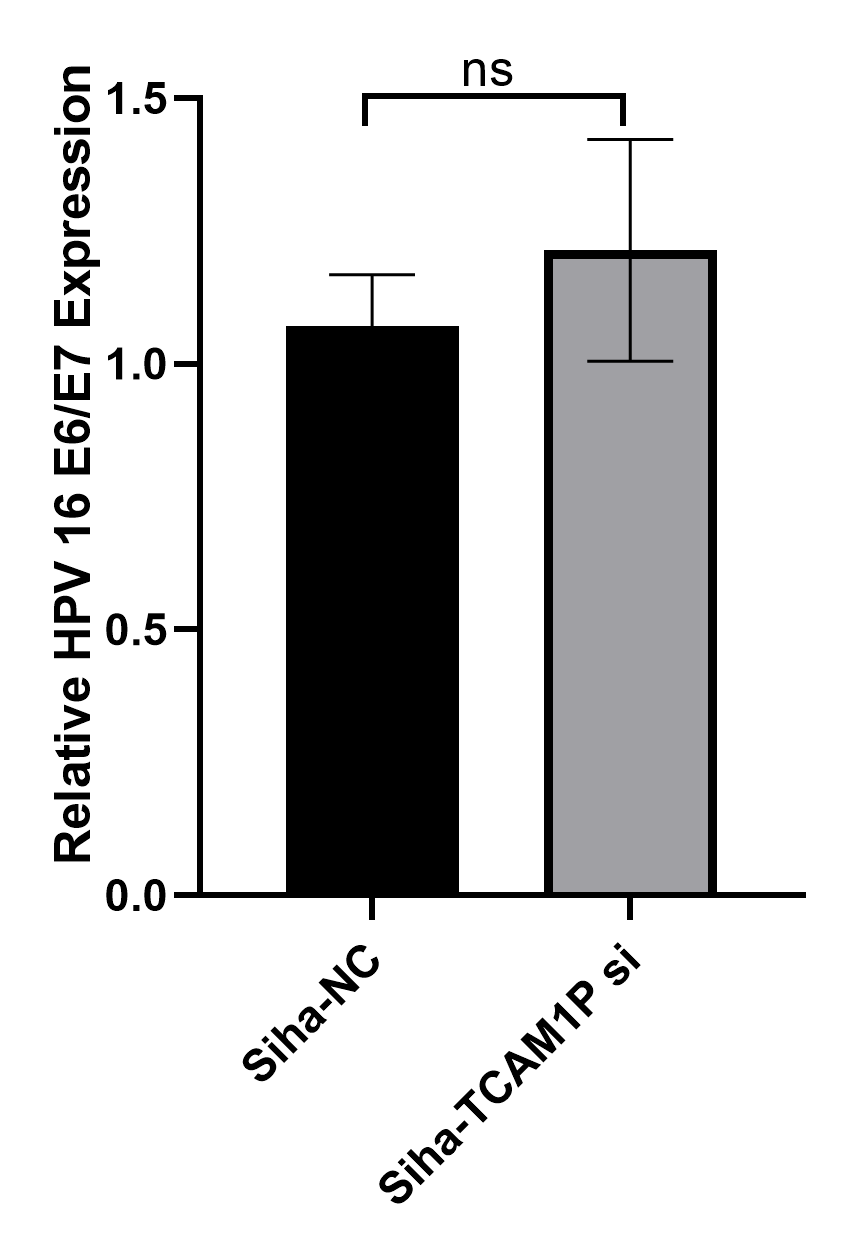

Supplement: Supplementary file 3 — Additional file 3. The expression of HPV 16 E6/E7 mRNA. [file 12935_2021_2440_MOESM3_ESM.tif]
